# Brown adipose tissue activity is modulated in olanzapine-treated young rats by simvastatin

**DOI:** 10.1186/s40360-020-00427-0

**Published:** 2020-06-30

**Authors:** Xuemei Liu, Xiyu Feng, Chao Deng, Lu Liu, Yanping Zeng, Chang-Hua Hu

**Affiliations:** 1 College of Pharmaceutical Sciences, Medical Research Institute, Southwest University, Chongqing, 400715 PR China; 2 Engineer Research Center of Chongqing Pharmaceutical Process and Quality Control, Chongqing, 400715 PR China; 3 School of Medicine and Molecular Horizons, University of Wollongong, Wollongong, NSW 2522 Australia; 4 Antipsychotic Research Laboratory, Illawarra Health and Medical Research Institute, Wollongong, NSW 2522 Australia; 5 North Sichuan Medical College, Nanchong, 637000 PR China

**Keywords:** Simvastatin, Olanzapine, Brown adipose tissue, Body weight gain, Dyslipidemia

## Abstract

**Background:**

Prescription of second-generation antipsychotic drugs (SGAs) to childhood/adolescent has exponentially increased in recent years, which was associated with the greater risk of significant weight gain and dyslipidemia. Statin is considered a potential preventive and treatment approach for reducing SGA-induced weight gain and dyslipidemia in schizophrenia patients. However, the effect of statin treatment in children and adolescents with SGA-induced dyslipidemia is not clearly demonstrated.

**Methods:**

To investigate the efficacy of statin interventions for reversing SGA-induced dyslipidemia, young Sprague Dawley rats were treated orally with either olanzapine (1.0 mg/kg, *t.i.d*.), simvastatin (3.0 mg/kg, *t.i.d*.), olanzapine plus simvastatin (O + S), or vehicle (control) for 5 weeks.

**Results:**

Olanzapine treatment increased weight gain, food intake and feeding efficiency compared to the control, while O + S co-treatment significantly reversed body weight gain but without significant effects on food intake. Moreover, olanzapine treatment induced a slight but significant reduction in body temperature, with a decrease in locomotor activity. Fasting plasma glucose, triglycerides (TG), and total cholesterol (TC) levels were markedly elevated in the olanzapine-only group, whereas O + S co-treatment significantly ameliorated these changes. Pronounced activation of lipogenic gene expression in the liver and down-regulated expression of uncoupling protein-1 (UCP1) and peroxisome-proliferator-activated receptor-γ co-activator-1α (PGC-1α) in brown adipose tissue (BAT) was observed in the olanzapine-only group. Interestingly, these protein changes could be reversed by co-treatment with O + B.

**Conclusions:**

Simvastatin is effective in ameliorating TC and TG elevated by olanzapine. Modulation of BAT activity by statins could be a partial mechanism in reducing metabolic side effects caused by SGAs in child and adolescent patients.

**Graphical abstract:**

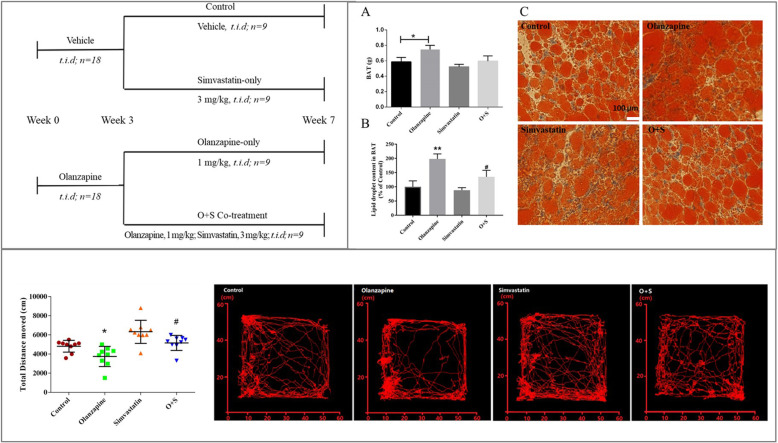

## Background

Prescription of second-generation antipsychotic drugs (SGAs) to childhood/adolescent has exponentially increased in recent years [1–3]. However, metabolic side effects are highly prevalent in schizophrenia patient treated with SGAs including olanzapine [4–6]. Several studies indicate that youth are more susceptible to metabolic side-effects of SGAs compared to adults [7–9]. In our previous studies, we demonstrated that SGAs markedly increase the expression of fatty acid- (such as *Fasn* and *Acc*1) and cholesterol biosynthetic genes (such as *Hmgcs* and *Hmgcr*, see for *Abbreviations* complete names) in liver, mediated via activation of the sterol regulatory element-binding proteins (SREBP1 and SREBP2) [10–12]. In general, SREBP1 controls the expression of fatty acid biosynthesis genes, while SREBP2 mainly regulates cholesterol biosynthetic genes. The SREBP-mediated activation of hepatic lipogenesis represents a new mechanism of psychotropic drug-induced the metabolic side-effects.

Decreased energy expenditure is also a key contributor to body weight gain and glucose-lipid metabolic disorder under chronic SGA treatments [13–16]. Brown adipose tissue (BAT), as an important player in lipid metabolism in rodents, mediates the process of adaptive thermogenesis and plays important roles in maintaining energy homeostasis [17]. BAT is rich in mitochondria with uncoupling protein 1 (UCP1) in the inner mitochondrial membrane, which uncouples the oxidative phosphorylation from ATP synthase, dissipating energy in the form of heat instead of ATP. UCP1 gene transcription is largely controlled through the cAMP-PKA signaling pathway by noradrenaline released from the sympathetic nerves, acting at β3-adrenergic receptors on the surface of brown adipocytes. Notably, UCP1 expression in BAT was reduced following chronic SGA treatment, leading to a lower response to cAMP stimulus [13–15, 18].

Statins, 3-hydroxy-3-methylgutaryl-COA (HMG-CoA) reductase inhibitors, are considered a potential preventive and treatment approach for reducing SGA-induced weight gain and dyslipidemia in schizophrenia patients. Atorvastatin [19], lovastatin [20], rosuvastatin [21], or simvastatin [22] were reported to lower TC, LDL-C and TG among dyslipidemic psychiatric patients. Statins were found an obvious method for lowering cholesterol and reducing risk in children with familial hypercholesterolemia [23, 24], although the effect of statins treatment in children and adolescents with SGA-induced dyslipidemia is not clearly demonstrated.

Statins are the most therapeutically effective as cholesterol-lowering agents targeting HMG-CoA reductase. It was reported that atorvastatin had an inhibitory effect on adipocyte differentiation which might contribute to pleiotropic actions of statins [25]. Additionally, atorvastatin treatment could accelerate the hepatic uptake of cholesterol-enriched lipoprotein remnants generated by BAT activation, thereby increasing the lipid-lowering and anti-atherogenic effect [26]. However, to date there has been little research elucidating whether co-treatment of statins could have lipid-lowering effects though intervening in BAT thermogenesis. Therefore, the present study investigated the effect of simvastatin in preventing olanzapine-induced weight gain and dyslipidemia in a young rat model. We propose that inhibition of SREBP-controlled HMG-CoA reductase activation represent an important statin-mediated mechanism of the improvement of dyslipidemia induced by SGAs, and the hypolipidemic effect of statin might be partly via activating the function of BAT.

## Methods

### Animals, housing and drug treatment

Young female Sprague-Dawley (SD) (45-55 g, 3 weeks old) rats were obtained from the Animal Resource Center (China), housed at 22 °C, on a 12 h light-dark cycle, and allowed ad libitum access to water and standard laboratory chow diet (3.9 kcal/g; 10% fat, 74% carbohydrate and 16% protein) for the duration of the experiment. After a one-week habituation, rats were trained to self-administer a sweet cookie dough pellet 0.3 g (30.9% cornstarch, 30.9% sucrose, 6.3% gelatin, 15.5% casein, 6.4% fiber, 8.4% minerals and 1.6% vitamins) without drugs for one week. All animal experiments were performed in accordance with the National Institute of Health Guide for the Care and Use of Laboratory Animals (Publication No. 85–23, revised 1985), and approved by experimental the Animal Ethics Committee of School of Pharmaceutical Sciences, Southwest University, Chongqing, China. Minimising the number of animals and their suffering was our general practice throughout this study.

**Fig. 1:**
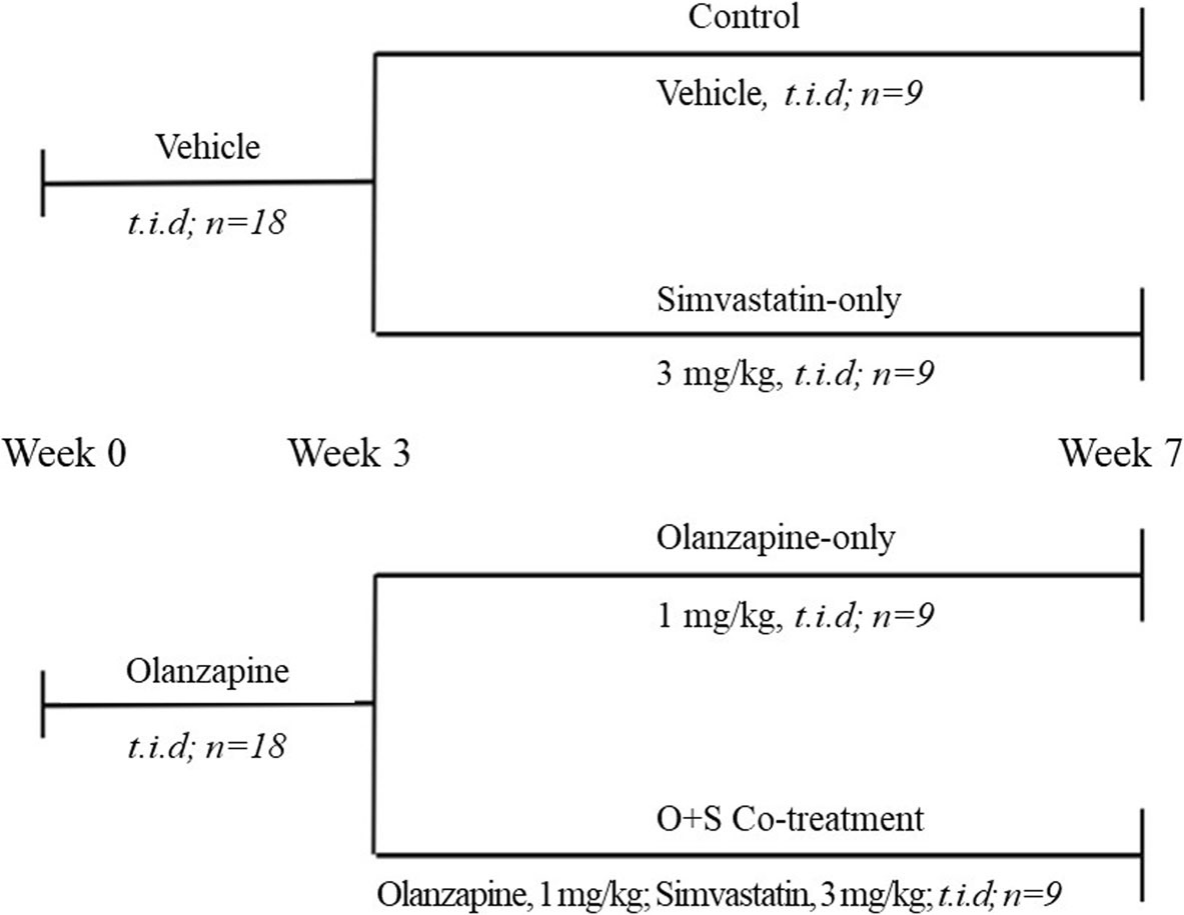
Outline of the experimental design

As shown in Fig. 1, 36 young rats were randomly assigned to two groups (*n* = 18/group): olanzapine (1 mg/kg, Eli Lilly, USA) three times per day (*t.i.d*.), and vehicle (*t.i.d*.) for 2 weeks treatment. The two groups were then divided into four subgroups (*n* = 9) for a further 5 weeks’ treatment: (1) control (received a sweet cookie dough pellet without drug, *t.i.d*.); (2) olanzapine-only (1 mg/kg, *t.i.d*.), (3) simvastatin-only (3 mg/kg, *t.i.d*., Merck, USA) and (4) co-treatment of olanzapine and simvastatin (O + S). Body weight, food intake and rectal temperature were measured once every 2 days throughout the experiment period. Blood was collected in EDTA tubes under ether anesthesia on the 14th day and 48th day, followed by centrifugation to isolate plasma which was stored at − 80 °C freezer until assay.

After 7 weeks’ of drug treatment, rats were individually housed and fasted for 10 h, and during the last 2 h of fasting, they were exposed to low temperature (16 °C). At the end of the cold exposure, blood was collected and interscapular BAT and liver were harvested, weighed and frozen in liquid nitrogen. These tissue samples were stored at − 80 °C freezer until assay.

### Measurement of rectal temperature of rats

Before rectal temperature was formally measured, the rats were adapted to the rectal probe (Yitainuo, Beijing) for 10 days. During the measurement, the environment was consistent with the rats’ living environment to avoid a stress reaction in the rats. The measurement was conducted gently at 9.30 am (2 h post treatment).

### Open field test

Locomotor activity contributes to energy expenditure. To determine whether olanzapine and/or simvastatin influenced the locomotor activity of rats, an open field test was carried out between 09:00 am and 17:00 pm. Every rat was placed in the center of a black rectangular area (50 × 50 cm^2^, 50 cm high) on the 43rd day of the drug treatment. The behavior of the rats was recorded from the top by a video camera for 25 min. Locomotor activity was analyzed by Noldus observer (Noldus Information Technology, Netherlands). Total distance moved (cm) was measured.

### Plasma assay

Triglyceride, total cholesterol and glucose concentrations were analyzed using the Assay Kits (Jiancheng, China), which were described in detail in the previous study [27].

### Oil-red-O staining

Frozen liver and BAT of rats were sectioned 10 μm thick using a cryostat section and fixed with 10% formalin for 30 min, respectively. Lipid droplets were detected by Oil-Red-O (ORO) staining (Sigma-Aldrich, USA). Sections were stained for 15 min in ORO solution and counterstained with hematoxylin (Sigma-Aldrich, USA) for 30 s. The images were photographed by inverted microscope (Olympus, Japan).

### Quantitative real-time PCR (qRT-PCR)

Total RNA was extracted from tissue samples using Trizol reagent (TianGen, China) and reversely transcripted for cDNA synthesis with a Transcriptor First Strand cDNA Synthesis Kit (Roche, Germany). Real-time PCR was performed with the SYBR Green PCR Master Mix (Applied Biosystems, USA). *Gapdh* and *β-actin* were used as the endogenous control.

### Western blot analyses

Protein samples were extracted from tissue homogenized in Radio Immunoprecipitation Assay (RIPA) buffer with Protease Inhibitor Cocktail (Dingguo, China). Aliquots containing 10 μg of proteins were loaded onto a 10% sodium dodecyl sulfate–polyacrylamide gel, transblotted onto a polyvinylidene difluoride membrane (Bio-Rad), blocked with 5% BSA in Tris-buffered saline with 0.1% Tween-20, and then incubated with the primary antibodies for UCP1 (1:1000, Santa Cruz, sc-6529), peroxisome proliferator activated receptor gamma (PPARγ) (1:1000, Santa Cruz, sc-6285), PPARγ coactivator 1-alpha (PGC-1α) (1:1000, Santa Cruz, sc-13,067), and PR domain containing 16 (PRDM16) (1:1000, Absin, abs104818), PKA (1:1000, cell signaling technology, 4782S), p-PKA (pThr197) (1:1000, cell signaling technology, 4781S) and β-ACTIN (1:2000, Santa Cruz, sc-47,778). Protein visualization was used and the electrochemiluminescence (ECL) detection reagents and films were exposed on the chemiluminescence imaging system (Tanon, China) analyzed using the Image-J. Relative protein expression was normalized with the expression level of β-ACTIN.

### Statistical analysis

All data were analyzed by the SPSS software (IBM version 17.0, SPSS Inc., USA). Data were analyzed for normal distribution by the Kolmogorov-Smirnov test. One-way ANOVAs were applied to analyze data of body weight, food intake, and rectal temperature from Week 0 and 2, as well as food efficiency, white and brown adipose tissue weight, locomotor activity, mRNA and protein expression. From Week 3 to 7, body weight gain, food intake and body temperature were analyzed by three-way repeated ANOVAs (OLANZAPINE×SIMVASTATIN×TIME as repeated factors). Pearson’s correlation test was used to analyze the relationships among the measurements. Multiple comparisons were performed using *post-hoc* Dunnett *t*-tests for comparing each drug treatment group with controls. A Mann-Whitney *U* test was applied for the data without normal distribution. Data were expressed as mean ± standard error of the mean (SEM), and statistical significance was accepted when *p < 0.05*.

## Results

### Body weight gain

**Fig. 2:**
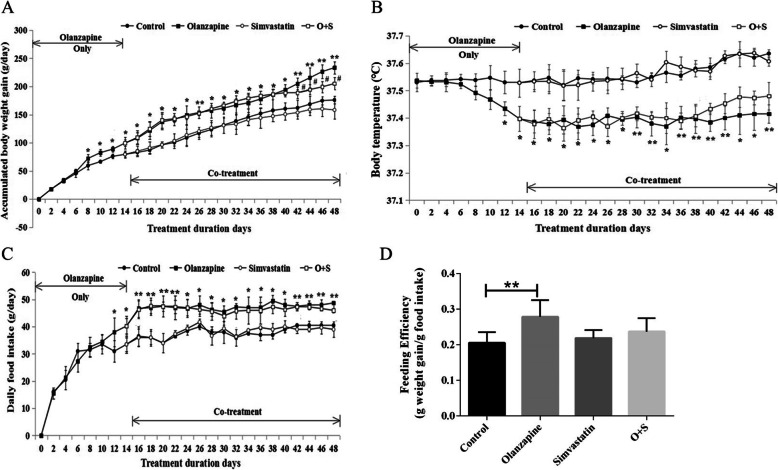
Effects of olanzapine and/or simvastatin treatment on (**a**) body weight gain, (**b**) body temperature, (**c**) food intake and (**d**) feeding efficiency of rats over the experiment period. Rats were administrated orally with olanzapine (1 mg/kg, *t.i.d*), simvastatin (3 mg/kg, *t.i.d*), co-treatment (O + S) or control (vehicle) for 7 weeks. Data are presented as mean ± SEM (*n* = 9 per group). *p < 0.05, **p < 0.01 vs. control, *#p <* 0.05 vs. olanzapine-only group. O + S, co-treatment with olanzapine and simvastatin

As shown in Fig. 2a, from Week 0 to Week 2, there was significant interaction between the TIME and OLANZAPINE factors (*F*_7, 238_ = 13.23, *p* < 0.001). Olanzapine treatment significantly increased body weight gain compared to vehicle treatment from day 8 (Fig. 2a, *p* < 0.05). Three-way repeated ANOVAs (OLANZAPINE × SIMVASTATIN× TIME as repeated measures) showed significant main effects of TIME (*F*_16, 512_ = 17.78, *p* < 0.001). There was a significant interaction between the OLANZAPINE and SIMVASTATIN factors (*F*_1, 32_ = 12.17, *p* < 0.05) for the last 5 weeks. As shown in Fig. 2a, in the olanzapine-only group, continuous olanzapine treatment significantly increased body weight gain compared to the control throughout the 5 weeks’ treatment (Fig. 2a, *p* < 0.05), whereas the O + S co-treatment group had a lower weight gain compared with the olanzapine-only treatment group after 4 weeks’ co-treatment (Fig. 2a, *p* < 0.05).

### Body temperature

Olanzapine treatment significantly decreased body temperature compared to vehicle from day 12 (Fig. 2b, *p* < 0.05). During the last 5 weeks, there was a significant interaction between the OLANZAPINE factor and TIME factor (*F*_16,512_ = 2.1, *p* < 0.05). Body temperature was still significantly lower in the olanzapine-only compared to the control (Fig. 2b, *p* < 0.05). It was interesting that the O + S co-treatment group increased body temperature at a borderline significance compared with the olanzapine-only group from day 40 (*p* = 0.083).

### Food intake and feeding efficiency

During the first 2 weeks (Day 0–14), there was a significant interaction between the TIME and OLANZAPINE factors (*F*_7, 238_ = 446.87, *p* < 0.001). Compared to the control group from day 12, a significant increase in food intake was observed in the olanzapine group (Fig. 2c, *p* < 0.05). From week 3 to week 7, the two groups were divided into four subgroups. In the olanzapine-only group, a significant increase in food intake was observed (*p* < 0.05). Moreover, feeding efficiency (grams of weight gained/grams of food consumed) was significantly elevated by olanzapine treatment compared with the control group (*p* < 0.05). However, no significant difference in food intake was detected between the O + S co-treatment group and olanzapine-only group (Fig. 2c). Furthermore, O + S co-treatment was not effective in decreasing feeding efficiency (grams of weight gained/grams of food consumed) compared to the olanzapine-only treatment (Fig. 2d).

### Fat deposits

**Fig. 3:**
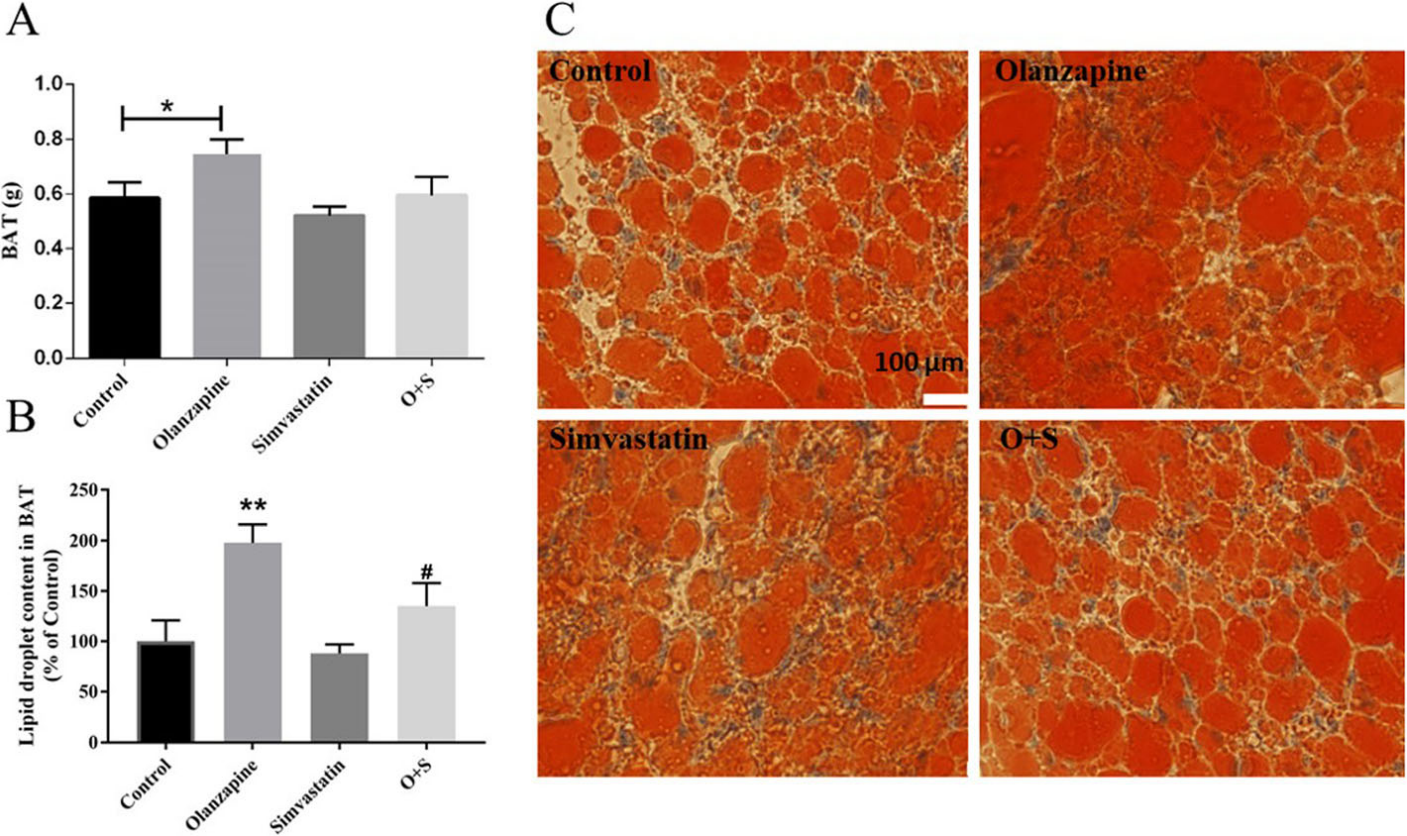
Olanzapine and simvastatin co-treatment reduced fat accumulation in the adipose tissue. **a** Mass of BAT; **b** Lipid droplet content in BAT; **c** Representative images of randomly selected sections of BAT stained for Oil red O in rats. Scale bars, 100 μm. Excessive lipid accumulation in BAT could indicate a phenotypic switch of BAT. O + S, co-treatment with olanzapine and simvastatin; BAT: brown adipose tissue. *p < 0.05 vs. control, *#p <* 0.05 vs. Olanzapine-only group

As shown in Fig. 3a, compared with control, BAT weight was significantly higher in olanzapine-treated rats (*p* < 0.05). Histological analysis of BAT revealed significant difference in adipocyte size or number between olanzapine-only group and control (Fig. 3b and c, *p* < 0.01). It is important that combination with simvastatin treatment reduced lipid droplet content in BAT (approx. -60%, *p* < 0.05), with a decrease of relative BAT weight as compared to olanzapine-only group (approx. -15%; *p* = 0.093, Fig. 3b and c).

### Locomotor activity

**Fig. 4:**
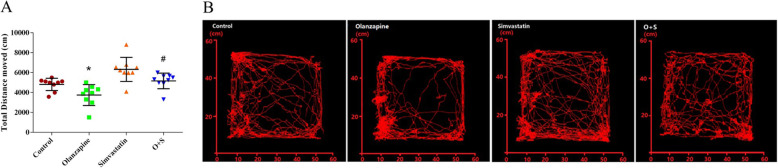
Effects of olanzapine and/or simvastatin treatment on locomotor activity. **a** Total distance moved in the open field test as at the 43rd day of olanzapine and/or simvastatin treatment. **b** Locomotor activity in the open field test was traced by Noldus observer. *p < 0.05, **p < 0.01 vs. control

There was a significant effect of the OLANZAPINE factor on distance moved (*F*_1, 32_ = 9.02, *p* < 0.05). The olanzapine-only group had significantly less distance moved than the control group (*p* < 0.05). It is important that the rats with simvastatin treatment had a significant increase in the total distance moved over the control group (*p* < 0.05) (Fig. 4a and b). There were negative correlations between total distance and body weight gain (*r* = − 0.334, *p* < 0.05). However, no significant difference in locomotor activity was detected between the O + S co-treatment group and olanzapine-only group.

### Serum biochemical parameters

As shown in Table 1, olanzapine led to higher levels of triglycerides, total cholesterol and glucose (all *p <* 0.05) than the control. When the two groups divided into four groups, the chronic olanzapine-only treatment further induced triglycerides, total cholesterol and glucose to remain at higher levels (all *p <* 0.05, Table 2). The simvastatin-only group significantly reduced triglycerides and total cholesterol compared to the control group (all *p* < 0.05, Table 2). Co-treatment of olanzapine and simvastatin reversed the levels of triglyceride, total cholesterol and glucose to normal levels when compared with the olanzapine-only group (all *p <* 0.01, Table 2). There was a positive correlation between body weight gain and triglycerides (*r* = 0.487, *p* < 0.01).

**Table 1:** Average triglyceride, total cholesterol and glucose levels (mmol/l) in the plasma on the 14th day of olanzapine treatment (*n* = 18)

Group	Triglyceride (mmol/l)	Total Cholesterol (mmol/l)	Glucose (mmol/l)
**Control**	0.73 ± 0.02	2.28 ± 0.03	7.39 ± 0.11
**Olanzapine**	1.02 ± 0.06^**^	2.40 ± 0.04^*^	7.74 ± 0.13^*^

*Abbreviations*: *p < 0.05, **p < 0.01 vs. control

**Table 2:** Average triglyceride, total cholesterol and glucose levels (mmol/l) in the plasma on the 48th day of olanzapine and/or simvastatin treatment (*n* = 9)

Group	Triglyceride (mmol/l)	Total Cholesterol (mmol/l)	Glucose (mmol/l)
**Control**	0.85 ± 0.06	2.45 ± 0.03	7.38 ± 0.02
**Olanzapine**	1.27 ± 0.04^**^	2.74 ± 0.06^**^	7.95 ± 0.18^*^
**Simvastatin**	0.59 ± 0.06^*^	2.22 ± 0.10^*^	7.35 ± 0.03
**O + S**	**0.84 ± 0.03**^**##**^	**2.43 ± 0.05**^**##**^	**7.47 ± 0.03**^**##**^

*Abbreviations*: O + S, co-treatment with olanzapine and simvastatin. *p < 0.05, **p < 0.01 vs. control, *##p <* 0.01 vs. olanzapine

### mRNA expression levels in the liver

**Fig. 5:**
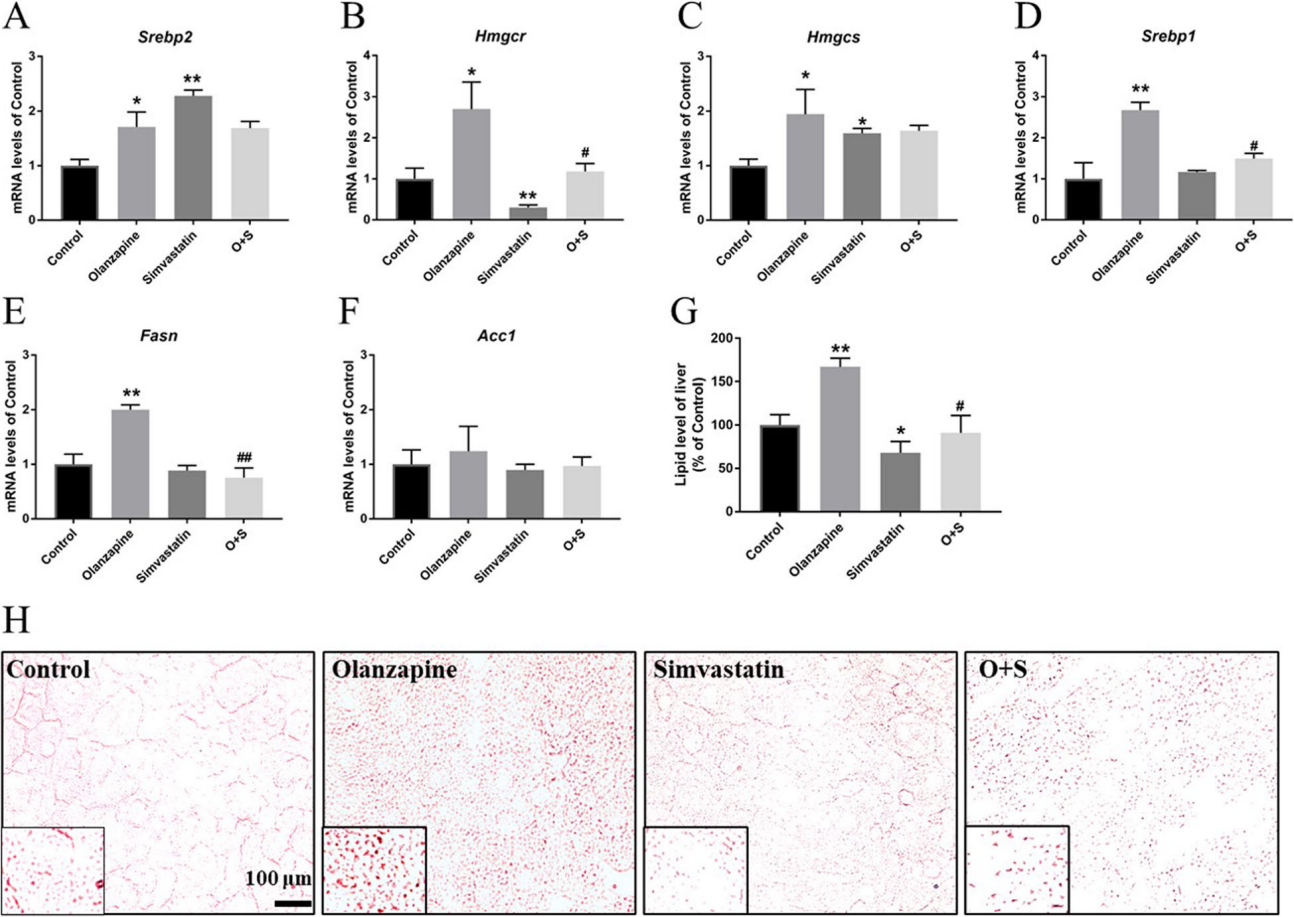
Effects of olanzapine, simvastatin treatment and simvastatin treatment combined with olanzapine on hepatic lipid levels. Hepatic mRNA expression of cholesterol biosynthesis and fatty acid synthesis-related genes: *Srebp2* (**a**), *Hmgcr* (**b**), *Hmgcs* (**c**), *Srebp1*(**d**), *Fasn* (**e**) and *Acc1* (**f**). **g** Lipid level of liver. The data from ORO staining. **h** Representative images of randomly selected sections of the liver stained for Oil red O in rats. Scale bars, 100 μm. Values are means ± SEM. *p < 0.05; **p < 0.01 vs. control; *#p <* 0.05, *##p <* 0.01 vs. olanzapine-only group

As presented in Fig. 5a, *Srebp2* (Fig. 5a, 1.71-fold increase, *p* < 0.05) and its target genes *hmgcr* (Fig. 5b, 2.70-fold increase, *p* < 0.05) and *hmgcs* (Fig. 5c, 1.94-fold increase, *p* < 0.05) were significantly up-regulated by olanzapine. In addition, there was also an upregulation of hepatic *Srebp*1 mRNA expression in olanzapine-treated rats compared to controls (Fig. 5d, 2.6-fold, *p* < 0.05). Consistent with the alteration of *Srebp*1, mRNA expression of *Fasn*, but not *Acc*1, was significantly increased by olanzapine treatment (Fig. 5e, 2.1-fold, *p* < 0.05). As an HMG-CoA reductase inhibitor, simvastatin significantly affected mRNA expression of *Hmgcr* (0.69-fold decrease, *p* < 0.01). Consequently, O + S co-treatment significantly reduced *Srebp*1, *Hmgcr* and *Fasn* transcriptional levels increased by olanzapine. However, an increase of *Srebp2* and *hmgcs* mRNA expression was observed in O + S co-treatment group. Histological analysis of liver revealed that olanzapine-only treatment significantly promoted accumulation of lipid droplets in the liver, whilst the O + S co-treatment decreased lipid droplets (Fig. 5g and h, *p* < 0.01). As shown in Fig. 5g and h, a significantly lower positive ORO staining was observed in the O + S co-treatment group than the olanzapine-only group (∼45.51% reduction, *p* < 0.01).

### Protein and mRNA levels of thermogenic gene in brown adipose tissue

**Fig. 6:**
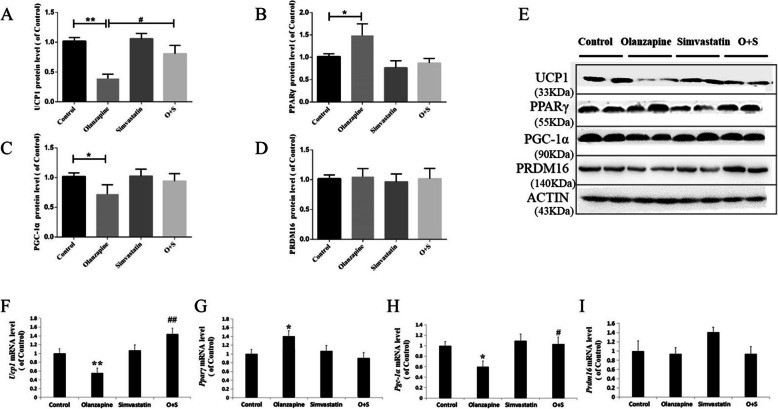
Effects of olanzapine and/or simvastatin treatment on protein levels of UCP1 (**a**), PPARγ (**b**), PGC-1α (**c**) and PRDM16 (**d**), and mRNA expression of *Ucp1* (**f**), *Pparγ* (**g**), *Pgc-1α* (**h**) and *Prdm16* (**i**) in BAT. The representative bands of Western blots are shown in (**e**). The data were normalized by taking the average value of the control group as 100% and expressed as mean ± SEM. *p < 0.05 vs. control, *#p <* 0.05, *##p <* 0.01 vs. olanzapine-only group

Compared to the control, olanzapine treatment dramatically decreased the protein levels of UCP1 (− 59%, *p* < 0.01, Fig. 6a and e) and PGC-1α (− 26%, *p* < 0.05, Fig. 6c and e) in the BAT, but not PRDM16. However, there was a significant increase in PPARγ expression in the olanzapine-only treatment group (+ 42%, *p* < 0.01, Fig. 6b and e). The O + S co-treatment significantly increased UCP1 expression compared with olanzapine-only treatment (+ 48%, *p* < 0.05, Fig. 6a and e). The UCP1 protein level was negatively correlated with body weight gain (*r* = − 0.516, *p <* 0.01).

Consistent with changes in protein levels, there was a significant decrease of *Ucp1* (− 45%, *p* < 0.05, Fig. 6f) and *Pgc-1α* (− 40%, *p* < 0.05, Fig. 6h) mRNA expression in the olanzapine-only group compared with the control. mRNA expression of *Pparγ* was significantly increased by olanzapine treatment compared with the control group (+ 40%, *p* < 0.05, Fig. 6g). Compared to the olanzapine-only group, O + S co-treatment upregulated *Ucp1* (+ 88%, *p* < 0.01, Fig. 6f) and *Pgc-1α* (+ 43%, *p* < 0.05, Fig. 6h) expression, but not *Pparγ* and *Prdm16* gene (Fig. 6g and i).

**Fig. 7:**
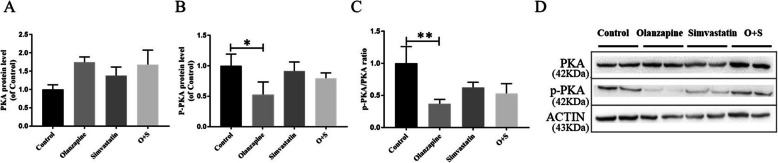
Olanzapine inhibits UCP1 expression via reducing the activation of the PKA pathway in BAT: PKA(**a**), p-PKA(**b**), the ratio of p-PKA/PKA(**c**) and (**d**) quantification of the bands. The data were normalized by taking the average value of the control group as 100% and expressed as mean ± SEM. *p < 0.05 vs. control, *#p <* 0.05, *##p <* 0.01 vs. olanzapine-only group

To further assess whether simvastatin could enhance the expression of UCP1 through the PKA-dependent pathway, we detected the expression of proteins of the cAMP-dependent protein kinase (PKA) and phosphorylated PKA (p-PKA). A decrease in p-PKA was observed in BAT from olanzapine-treated rat (− 48%, *p* < 0.05, Fig. 7b and d), although we did not detect a significant increase of proteins related to the PKA signaling pathway between the O + S co-treatment and the olanzapine-only groups.

## Discussion

In the past two decades, a number of clinical trials have proven that SGAs administration (particularly olanzapine and clozapine) causes significant weight gain and dyslipidemia in childhood/juveniles [3]. Consistent with clinical reports, this study revealed that olanzapine (1 mg/kg, *t.i.d*) led to a significant increase in body weight gain, BAT mass, food intake, feeding efficiency, and that it elevated the circulating triglycerides, total cholesterol and glucose in young rats. Our data further revealed that olanzapine treatment reduced locomotor activity and body temperature, furthermore it down-regulated protein levels and transcriptional expression of the crucial thermogenic genes involved in UCP1 and PGC-1α. In addition, we found that co-treatment with simvastatin improved olanzapine-induced dyslipidemia, inhibited transcriptional levels of *Hmgcr, Srebp1 and Fasn*, and reversed the decreased levels of UCP1 and PGC-1α in BAT by olanzapine treatment. The results suggest that, besides its well-known effects in targeting “HMG-CoA reductase”, simvastatin co-treatment may ameliorate olanzapine-induced dyslipidemia through inhibiting SREBP-controlled HMG-CoA reductase activation, and modulating the transcriptional responses of thermogenic genes, at least in part, to increase energy expenditure via upregulation of UCP1.

Consistently with previous studies, our results showed that olanzapine treatment significantly increased body weight gain, total cumulative food intake and enhanced feeding efficiency [13, 28]. Food intake was positively correlated with body weight gain, suggesting weight gain may be due to enhanced energy intake [29]. Our findings further confirmed that olanzapine treatment reduced voluntary locomotor activity and body temperature [14, 18]. However, Lord et al. found that olanzapine increased energy expenditure when mice were fed a compounded high-fat diet [30]. It is well known that BAT plays a crucial role in maintaining energy homeostasis in response to cold temperature and excess nutrition (adaptive thermogenesis). Therefore, a high fat diet could stimulate adaptive thermogenesis of mice [31, 32]. Future studies should elucidate whether the different eating habits could alter different aspects of energy homeostasis under SGAs administration. Importantly, the accumulation of lipid droplets and decreased protein levels of UCP1 and PGC-1α in BAT were observed in the olanzapine-only group, suggesting a reduction of BAT activation. It is known that UCP1 gene is directly regulated by PRDM16, which can be recruited to the UCP1 gene enhancer through interactions with PGC-1a [33]. In addition, the thermogenic activities could be stimulated through the cAMP-PKA signaling pathway by β-adrenergic receptors–mediated activation [34]. Olanzapine-only treatment greatly inhibited PKA phosphorylation in our study, which could down-regulate the protein expression of UCP1 and further reduce the thermogenic ability of BAT. Data from the present study have further revealed that reduced energy expenditure, in particular thermogenesis and locomotor activity, could contribute to sustained body weight gain caused by long-term SGA treatment, especially in the childhood/adolescent period [35].

Currently available statins, lovastatin, simvastatin, fluvastatin, atorvastatin, and rosuvastatin have been approved for children≥10 with familial hypercholesterolemia by FDA [36, 37]. Simvastatin is offered as a disintegrating oral tablet, which may be of use in the pediatric population. Therefore, following the FDA guideline, 3.0 mg/kg (t.i.d) of simvastatin was chosen for co-treatment with olanzapine in present study (≈30 mg of drug for a patient in 1 day) [38]. Young rats that received simvastatin-only treatment for 34 days showed nonsignificant changes in body weight gain, food intake and body temperature when compared with the control group. It was worth noting that co-treatment with simvastatin might reduce body weight gain induced by olanzapine treatment. In clinical studies, statins were associated with a very small reduction in body weight [39, 40], but a significant decrease in triglycerides, total cholesterol, LDL cholesterol, and non-high-density lipoprotein (non-HDL) cholesterol was observed [21]. HMG-CoA reductase is the target of statin therapy. We confirmed previous findings that simvastatin decreased mRNA expression of *Hmgcr* in the liver, consequently lowered plasma TC levels [41, 42]. In addition, co-treatment with simvastatin further reduced dyslipidemia development in liver through down-regulating mRNA expression levels of fatty acid synthesis-related genes, such as *Srebp1* and *Fasn*. It was interesting that a significant influence on olanzapine-induced inhibition of BAT thermogenesis was observed in O + S co-treatment group, especially the protein expression and the transcriptional levels of UCP1 and other core regulators of browning (PGC-1α), which was relevant with reduced weight gain and increased energy expenditure [43]. Our data showed that UCP1 protein levels were negatively correlated with body weight gain (*r* = − 0.516, *p <* 0.01). Thus, we hypothesize that simvastatin might impact on the thermogenesis of BAT to further improve lipid metabolic disorder. Surprisingly, the effects of co-treatment on body temperature was not detected from initial interventional treatment with simvastatin, it only occurred after 4 weeks’ co-treatment. Although fatty acids provide the main fuel for BAT thermogenesis, there is a high glucose requirement to maintain the Krebs cycle [44]. Since O + S co-treatment did not effectively reduce plasma glucose levels, this could explain why the thermogenic effect of simvastatin was causing body temperature to rise slowly.

Our study has some limitations. First, only the female rats were used. Clinically, female patients have a much higher risk than males for SGA-induced weight gain and other metabolic side-effects [45–47]. Endocrine factors may influence gender specificity of metabolic adverse effects caused by antipsychotics. For instance, the ovarian hormone estradiol plays an important role in olanzapine-induced hyperphagia in female rats [48]. Moreover, the olanzapine-induced weight gain model has been consistently established and validated in female rats in our and other laboratories [28, 29, 49, 50]. However, a recent report showed that female mice were completely protected against acute olanzapine-induced hyperglycemia [51]. Many factors would be involved in the effects of olanzapine on the balance of energy, including gender, race, even species. Second, we did not detect whether simvastatin treatment would alter the clearance/degradation of olanzapine in this study. Although a few meta-analyses clarified that adjunctive therapy with statins could improve psychiatric symptoms, either negative symptoms or positive symptoms [52], there was drug-drug interactions between combination of antipsychotic with cardiovascular medications used in schizophrenia [53]. Particular attention should be paid to evaluate harmful interactions between antipsychotics and cardiovascular medications. Also, in our study, housing of rat below their thermal neutral zone (29–31 °C) could result in activation of thermogenesis to defend their core temperature, which limits the clinical translatability of the results [54, 55]. It is a limitation although there was a control housed at room temperature (22 °C). It could be more consistent with human biology when rats are housed at thermoneutrality.

## Conclusions

In summary, simvastatin could potentially help to ameliorate metabolic abnormalities associated with long-term olanzapine treatment. The hypolipidemic effect of simvastatin might be partly via activating the function of BAT. These findings support a potential mechanism of simvastatin in ameliorating olanzapine-induced weight gain through mediation of energy expenditure. Due to a high risk of interactions and related adverse effects, particular attention should be paid while using cardiovascular medications with antipsychotics [53, 56]. Future studies evaluating a combination of atypical antipsychotics with statins may help to tilt the balance of benefit over risk ratio in favor of greater benefits with currently prescribed antipsychotics.

## Supplementary information

**Additional file 1.** Original picture--Fig. 6 revised.

**Additional file 2.** Original picture--Fig. 7 revised.

## Data Availability

All data generated or analyzed during this study available from the corresponding author on reasonable request. We declared that materials described in the manuscript, including all relevant raw data, will be freely available to any scientist wishing to use them for non-commercial purposes, without breaching participant confidentiality.

## Abbreviations

SGAs: Second-generation antipsychotic drugs
O + S: Olanzapine plus simvastatin
TG: Triglycerides
TC: Total cholesterol
UCP1: Uncoupling protein-1
PGC-1α: Peroxisome-proliferator-activated receptor-γ co-activator-1α
PPARγ: Peroxisome proliferator activated receptor gamma
PRDM16: PRD1-BF1-RIZ1 homologous domain containing 16
PKA: cAMP-dependent protein kinase
p-PKA: Phosphorylated PKA
HMG-CoA: 3-hydroxy-3-methylgutaryl-COA
BAT: Brown adipose tissue
SD: Sprague-Dawley
ORO: Oil-Red-O staining
ECL: Electrochemiluminescence
SEM: Standard error of the mean
SREBP: Sterol regulatory element binding protein
*Fasn*: Fatty acid synthetase
*Acc1*: Acetyl-CoA carboxylase
*Hmgcs*: 3-hydroxy-3-methylgutaryl-COA (HMG-CoA) synthase
*Hmgcr*: 3-hydroxy-3-methylgutaryl-COA (HMG-CoA) reductase
