# Supplementary figures and images for: Brown adipose tissue activity is modulated in olanzapine-treated young rats by simvastatin
Source: BMC Pharmacol Toxicol. 2020 Jun 30;21:48. doi: 10.1186/s40360-020-00427-0 (PMC7325271; doi:10.1186/s40360-020-00427-0)

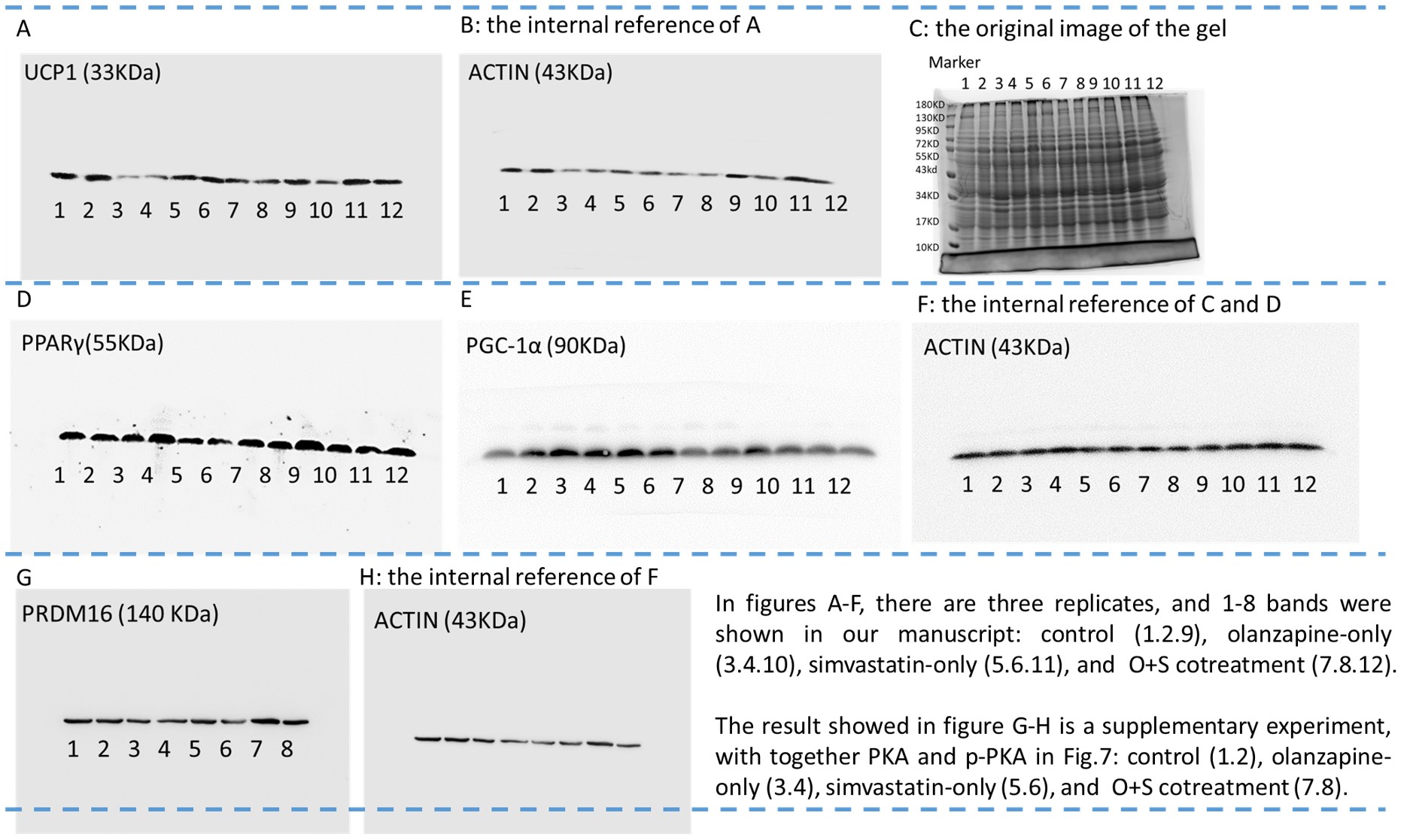

Supplement: Supplementary file 1 — Additional file 1. Original picture--Fig. 6 revised. [file 40360_2020_427_MOESM1_ESM.jpg]

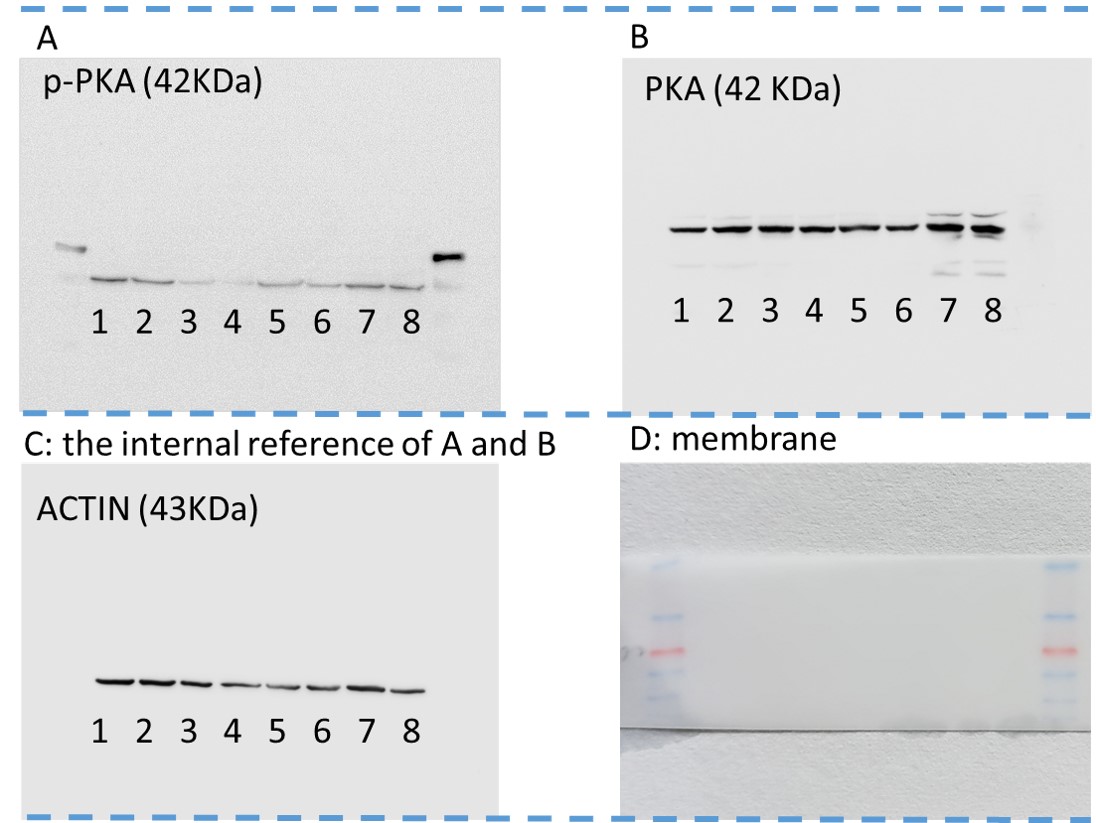

Supplement: Supplementary file 2 — Additional file 2. Original picture--Fig. 7 revised. [file 40360_2020_427_MOESM2_ESM.jpg]
